# Information and communication technologies for informal carers and paid assistants: benefits from micro-, meso-, and macro-levels

**DOI:** 10.1007/s10433-015-0333-4

**Published:** 2015-01-24

**Authors:** Stephanie Carretero, James Stewart, Clara Centeno

**Affiliations:** 1 Institute for Prospective Technological Studies, Joint Research Centre - European Commission, Edificio EXPO, c/ Inca Garcilaso, 3, 41092 Seville, Spain; 2 School of Social and Political Science, University of Edinburgh, Old Surgeons’ Hall High School Yards Edinburgh, Edinburgh, UK

**Keywords:** Quality of life, Quality of care, Efficiency, Cost-effectiveness, Long-term care, Social investment

## Abstract

The aim of this study was to explore the benefits of information and communication technologies (ICT)-based services for informal carers and paid assistants of older people living in the community. We cross-case analysed the effects of twelve initiatives in the EU, the USA and Canada, based on their individual analysis documented through interviews with promoters and a literature review. We carried out the cross-case analysis following a variables-oriented strategy on seven dimensions of impact at micro-, meso- and macro-levels: the quality of life of informal carers and paid assistants, quality of life of care recipients, quality of care, care efficiency and sustainability, acceptability, and infrastructure and accessibility. ICT-based services for informal carers and paid assistants improve the quality of life of older people and their carers and access to qualified care. They also generate savings which contribute to the sustainability of the care systems. These findings constitute a first look at the benefits of the use of ICT-based services for informal carers and paid assistants. Nevertheless, more research using experimental methods is needed to demonstrate the impact of these ICT-based services at meso- and macro-levels. This would help to support policy-makers to deploy these technologies for long-term care delivery.

## Introduction

This is a review of documentation from the project “Study on ICT-based solutions for caregivers: assessing their impact on the sustainability of long-term care in an ageing Europe” (CARICT), which ran throughout 2011. CARICT was aimed to find out the extent to which information and communication technologies (ICT)-based services for informal carers and their paid assistants exist in European countries, their impacts and the policy support needed for their development and implementation (Carretero et al. 2012).

Informal carers are defined as relatives, friends and neighbours, who care for older adults but are not trained or paid to provide care (Naiditch et al. 2013). In 2009, 3 % of persons in the EU-27 cared for a relative several times a week (Anderson et al. 2009), and the value of their care was estimated to range from 50 to 90 % of the overall costs of long-term care (Triantafillou et al. 2010). Research acknowledges that informal carers can be burdened by their care activities, and that their health, as well as their social, labour and economic life may be negatively affected (Carretero et al. 2009).

European governments have put in place some formal supports, mainly financial measures and in-kind services, to help informal carers to compensate for their economic loss and to allow them to reconcile care and work (Naiditch et al. 2013). Nevertheless, these solutions seem to only partially cover their needs: for example, some 50 % of families caring for older relatives are not satisfied with the public services available for families (Eurobarometer 2007). Moreover, in several European countries, in order to fill the gap not covered by formal services, some families resort to privately paid assistants, usually undeclared migrants who lack proper training to care for older people (Fujisawa and Colombo 2009; Kluzer et al. 2010).

Empirical evidence shows that informal carers and paid assistants have a wide range of social and care needs: psychological support; training and education; information, advice and counselling on caring; self-support; social participation with friends and peer groups; leisure activities; reconciliation of care and work; support in language and culture integration; and protection of their rights (Carretero et al. 2007, 2009; Barbabella et al. 2011). In response, information and communication technologies (ICTs) have been recognized by European policy as proactive measure to be developed in Member States to help in supporting the carers so that their older care recipients can be cared for at home (Social Protection Committee and European Commission 2014). ICT-based services for informal carers and paid assistants can be defined as a service provided by any private or public organisation that addresses some carers’ and/or care recipients’ needs through technological devices that are integrated or not in a wider intervention programme (Barbabella et al. 2011).

According to Kluzer et al. (2010) and Carretero et al. (2012), available ICTs to address carers’ needs include: (a) technologies that allow older people to stay at home without continual care support, relieving pressure on carers; (b) tools that give remote access to information and training about caring-related issues, such as websites and online training materials; (c) personal support and social integration that provide social, emotional and peer support, social networking systems for peer support and volunteer call networks (mobile, internet); and (d) online tools for coordinating care tasks from formal sector and informal and family employed carers, respite, and information sharing Examples of ICT for independent living (item a above) include home alarm systems, telecare, tracker devices (GPS), and (gas, temperature, bed) sensors. These technologies can give more independence to older people and their informal carers, as well as easing the constant worry of the latter (Billings et al. 2013). ICT services for information and learning (item b above) allow carers to access online and telephone-based information and advice from peers and professionals (Wu et al. 2009; The Princess Royal Trust for Carers 2012). Such ICTs also open up the possibility of online self-training through e-learning platforms for paid assistants to acquire skills or gain recognition for the skills they already have in managing long-term diseases (Pierce et al. 2004; Weiner et al. 2005). ICT services for support and integration (item c above) help the carers to maintain online contact with family and friends, to create online communities and to exchange information, advice and peer-support informally among themselves. For example, a study in the United Kingdom showed that the 42 % of online carers said that the Internet helps them reduce their feeling of isolation (The Princess Royal Trust for Carers 2012). Finally, an example of ICT for care coordination (item d above) may be seen in online organizational systems with carers’ access. These services sustain communication, coordination and cooperation with all those involved in care, and provide the possibility of arranging services online (Kluzer et al. 2010; The Princess Royal Trust for Carers 2012).

These technology-based services provide different kinds of support to address carers’ needs, specifically: (a) supporting older people with functional limitations; (b) ensuring quality of informal care; (c) supporting carer’s quality of life; (d) facilitating participation in aspects of life outside the home; and (e) enhancing the work done by the paid assistants of informal carers (Barbabella et al. 2011). However, ICT-based services for informal carers are not widely available on the market (Billings et al. 2013). For example, although ICTs for independent living such as the first generation of telecare and assistive technologies are already established in some countries, there are strong differences between or within countries (Carretero et al. 2012; Billings et al. 2013). Moreover, the use of ICT-based services such as smart homes is far from mainstreaming in most countries (Empirica and WRC 2010). The private and public sectors are reluctant to invest in developing or implementing these technologies, mostly because they lack demonstrated scientific evidence, beyond that of sundry pilot investigations of the consequences of the adoption of ICT-based services at the carer level as well as at the service and the welfare systems levels (Carretero et al. 2012).

Until now, the analysis of the benefits of ICT-based services has been mainly focused on the impact of these services at the micro-level, that is, at the elder/carer dyad. Some studies report that ICT-enabled services save carers’ time and money (The Princess Royal Trust for Carers 2012), increase their wellbeing (Smith and Toseland 2006; Lam and Lam 2012) and self-esteem (Weiner et al. 2005), help link them with others (Torp et al. 2008) and with the healthcare team (Shaw et al. 2004), and empower them, (Weiner et al. 2005) thus developing their problem solving ability (Buis 2008), perceptions of self-efficacy (Weiner et al. 2005; Gallagher-Thompson et al. 2007) and care skills (Weiner et al. 2005; Buis 2008). For example, Lam and Lam (2012) showed improved mental health among carers who were using Internet at the moment of the interview compared to those who were not. Wu et al. (2009) concluded that psychological interventions by phone and Internet can reduce anxiety, depression, and feelings of incompetence and increase disease knowledge of carers of patients with Alzheimer’ Disease. Pot et al. (2012) showed that carers felt less worried after 3 months of using tracking devices for people with dementia.

Nevertheless, the effects of ICTs for carers at the organizational (meso-) and system (macro)-levels are scarcely documented and have varying degrees of evidence (Morris et al. 2012; Billings et al. 2013). Data on impacts at the meso- and macro-levels could be relevant and complementary to those at the micro-level to convince policy-makers to promote policy frameworks for the creation of ICT-based services for carers, especially insofar as they provide data on the use of health and social services and savings. For this reason, the purpose of this review is to explore the impact of ICT-based services for informal carers and their paid assistants at the micro-, meso- *and* macro-levels, based on a cross-case analysis of documented impacts of implemented services in the EU, the USA, and Canada.

## Design and methods

This paper is based on the CARICT project which analysed ICT-based services for informal carers and their paid assistants using a method centred on (1) mapping existing ICT-based services to assist carers of older people living in the community in the EU, (2) defining a first prototype of impact-assessment methodology at the micro-, meso- and macro-levels and (3) analysing their success factors for policy recommendations (Carretero et al. 2012). The methodology used to explore the impacts of the ICT-based services at the respective levels focused on a cross-case analysis of these services, based on the mapping and on an impact-assessment methodology together with the identification of impacts carried out in CARICT.

The mapping consisted of a selection of a sample of ICT-based initiatives for informal carers and paid assistants in the EU Member States representing the five care regimes as identified in the literature: the Scandinavian, Anglo Saxon, Eastern European, Mediterranean and Continental care regimes (Simonazzi 2009) as well as geographical regions (3 initiatives per countries), reflecting the functions for carer activities and types of technologies (as defined in the first section), contingent on their being in operational status and with impact assessment being available (see Carretero et al. 2012). The mapping was done by reviewing previous national overviews and local studies (e.g. Empirica and WRC 2010; Kluzer et al. 2010; Yeandle and Fry 2010; Mollenkopf et al. 2010), and by face-to-face or telephonic interviews with at least four experts in the field from the five care regimes to solicit recent initiatives (such as those not available in English)—see Schmidt et al. (2011) for a list of experts and detailed information on the mapping method. As a result of these efforts, a sample of 52 initiatives was obtained (Carretero et al. 2012).

In parallel, a prototype of impact-assessment methodology was produced in order to be able to identify the multidimensional effects of ICTs in this context. The methodology was developed from a literature review of impact-assessment methodologies for home care, enriched by theories and practical tools, with a specific focus on the ICT role, and validated by experts (see Barbabella et al. 2011). The impact-assessment methodology identifies seven dimensions of impact of ICTs on informal carers in the home care context: quality of life of informal carers and paid assistants; quality of life of care recipients; quality of care; care efficiency and sustainability; acceptability; and infrastructure and accessibility. The seven dimensions were connected with indicators that measure these impacts according to three levels of analysis at micro (impact on carers and care recipients)-, meso (impact on families, companies and organizations, peer groups and care providers)- and macro-levels (impact on the economic and social protection systems, and the health and social care systems). A definition of the dimensions and examples of indicators by dimension can be found in Table 1 (Barbabella et al. 2011).

**Table 1:** Dimensions and examples of indicators at micro-, meso- and macro-levels of the impact-assessment methodology of ICT-based initiatives

Dimensions		Indicators		
		Micro	Meso	Macro
Quality of Life of Informal Carer	Reconciliation between care and work (possibility to keep the job while caring, to enter into the job market and to improve the work condition)	Possibility to balance well care & work activities	Efficiency at work	Number of carers that balance care & work activities
	Social life (aspects related to carers’ social relationships and participation, and their interactions with significant others)	Positive social contacts & relationships	Reduction in family conflicts	Increased social cohesion & inclusion
	Other dimensions of quality of life (health.)	Psychophysical health & life satisfaction	Number of non-stressed carers	
Quality of life of paid assistant (health of the carer, its prerequisites and effects, as well as the social life and work-related aspects).		Psychophysical health and independence level	Number of non-stressed carers	
Quality of life of care recipient (health of the care recipient, its prerequisites and effects, as well as their social relationships)		Physical level; Psychological level; Independence level	Number of reported cases of abuse/neglect by family members	Target number of dependent older people supported
Quality of care (necessary prerequisites and the outcomes of the care provided by carers)		Improvement of caring activities by direct (e.g. training) or indirect (e.g. decreasing burden of carer) factors		
Care efficiency & sustainability (economic sustainability of the initiative and the efficiency in terms of costs and quality of the final outcome)		Care efficiency (in terms of quality and cost containment) and sustainability for care recipients and families	Efficiency and Sustainability for care providers	Efficiency and Sustainability for Social Protection and Care systems
Acceptability (aspects making the initiative attractive towards the main actors at all levels)		Acceptability by Carer and care recipient	Marketability of ICT devices (from ICT device producer point of view)	Resources of Care system to support ICT devices (e.g. public investments in ICT)
Infrastructure & accessibility (the impact of infrastructure facilities on the access and use of ICT-based services)		Accessibility of initiative by carers	Availability of services (from care provider point of view)	Availability of services (from system point of view)

The sample of 52 initiatives was used as the source for the selection of a heterogeneous sample of initiatives that could allow us to appropriately analyse the impact of the services. We then carried out a pre-selection of initiatives, taking into account that to be very diverse so as to ensure a range of support functions and types of ICT (as defined in the introduction) (Schmidt et al. 2011). Twenty initiatives were first pre-selected, of which 10 ICT-based services representing a balance among the different care regimes were finally selected to be part of the sample for impact analysis. This sample was complemented by the addition of two initiatives from North America, as they constituted recognized examples of high-quality impact-assessment design with convincing evidence of positive outcomes (Available in Schmidt et al. 2011). Thus, our sample consisted of twelve ICT-based services (Table 2)—see Chiatti et al. 2011 for more details on the service.

**Table 2:** Overview of the sample of the 12 selected ICT-based initiatives

Name	Start year	Country	Articles/docs	Service description	Typology of technological service			
					ID	I & L	PS & SI	CC
Action	1998	Sweden	20	Older spouse carers can use Web-based training support, video phone links to a social care call centre, to communicate with network of families and practitioners		X	X	X
Campus	2004	Italy	3	Set of online and DVD-based training material for informal carers to improve caring skills and life chances for families, carers and migrant care assistants		X	X	
Caring for others	2000	Canada	10	10 weekly group training sessions via internet video to high burden housebound carers, with follow-up video support group, and online information		X	X	
Cuidadoras en red	2008	Spain	2	Training courses on ICT-based skills to improve access to information and social support, primarily for family carers and care assistants. Online community		X	X	
E-care	2005	Italy	9	Care planning and coordination with multiple agencies, families and volunteers; provides call centres, telealarms, telehealth, video conferencing, online information, telefriending	X	X	X	X
Ippi + Amigo	2004	Sweden	5	TV-based communication system for older people to communicate with care services and family with call centre for relatives to update and coordinate care	X	X	X	X
Emergency alarm	1994	Hungary	2	Social alarm over GSM, with call centre staffed primarily by volunteers, facilitating home care	X			
Just checking	2003	UK	6	Electronic Monitoring of movements of early-stage dementia sufferers living at home helps professionals and family better understand care needs, building trust, and facilitating independent living and home care	X	X		
Platform for caring family members	2006	Austria	2	Information web site and hot line in two languages about caring, services etc.		X		
Reach I/II	1995, 2001	USA	4	Integrated service supported by nurses, online, video and telephone including therapy, advice, a bulletin board and training which aims to reduce the burden and depression in family carers, to support carers of dementia sufferers		X	X	
Sophia	2005	Germany	2	Multiple types of social alarm, and a call centre to support identification of older people’s needs, and provide phone-based social support to older people and carers from volunteers	X			
Telecare scotland	2006–2011	UK	11	Social alarm and home care sensors to help local care services and family members care for older people in the community	X		X	X

Articles/docs: number of articles/documents reviewed.* ID* ICT for independent living.* I & L* ICT for information and learning.* PS & SI* ICT for personal support and social integration.* CC* ICT for care coordination

The impact of these twelve ICT-based services was individually analysed using the impact-assessment methodology developed specifically for this purpose (Barbabella et al. 2011). Concretely, a case study method was used to analyse each of the 12 initiatives, using a common template to report the case information (Chiatti et al. 2011). The case study method documented the evaluation methods and all the impact-assessment and evaluation data available from each service including references to the original studies, in order to show the actual impact of the service as it was measured. Data collection was carried out in the period from April to October 2011 (last data update), through a small number of face to face or phone interviews with initiative coordinators and project managers of the services, as well as web-based research and secondary analysis of existing data. We followed this up with a cross-case analysis of the impact of the twelve relevant initiatives. For this specific paper, we carried out a cross-case analysis of the individual analysis of the twelve ICT-based services, following a variables-oriented strategy (Miles and Huberman 1994) according to the variables defined in the prototype of impact-assessment methodology.

## Results

This section describes the results of the cross-case analysis of the impact of the 12 relevant ICT-based services for informal carers and paid assistants, at the micro-, meso- and macro-levels.

### Impact at micro-level (impact on carers and care recipients)

Regarding the quality of life of informal carers, we found that ICTs for independent living can indeed help informal carers to reconcile care and work. The ICT-based services aid carers to remain in paid employment despite their having to care for a dependent older person. This is an indirect consequence of the relief offered by services such as telehealth and telecare systems to informal carers. For example, interviews with 18 family carers using the “Emergency Alarm” service in 2011 revealed that they perceived that they better balanced care with work after the installation of the system. They also reported having fewer health problems and more leisure time and relief (Chiatti et al. 2011).

ICTs for personal support and social integration clearly improve carers’ social lives. Social tools (e.g. online forums, video-chats) that address both personal and social integration needs assist the informal carers to reduce their social isolation as well as improving their social activities and intergenerational relationships. For example, the “Action” initiative in Sweden reduced informal carers’ social isolation, increased their sense of security and improved their subjective quality of life (Magnusson et al. 2002; Bergström et al. 2010), and the “Cuidadoras en Red” service in Spain improved social activities and intergenerational relationships (Carrión and Armayones 2006). The “IPPI” initiative in Sweden facilitated communication between relatives and healthcare providers, making it possible to plan better (Östlund and Lindén 2011).

ICT-based services for informal carers also improved their health. The main improvements reported were: better physical and mental health, less burden, better emotional condition, fewer depressive symptoms and a certain amount of relief from sorrow. These results are seen to be the direct effect of informal carers’ use of technologies for personal support and social integration and the indirect impact of other technologies such as those for care coordination. For instance, these ICTs allow families to coordinate and communicate better with health professionals and therefore to integrate home care with care services more effectively. This in turn leads to less stress and more relief for carers, as shown by the “Action” (Bergström et al. 2010), “IPPI” (Östlund and Lindén 2011) and “Just Checking” (Warwickshire County Council 2006) initiatives. The service “Caring for Others” in Canada was related to informal carers’ better physical and mental health, and a reduction in the stress associated with caring (Marziali and Garcia 2011). ICT-based interventions under the “REACH” programs in the United States obtained significant improvements in carers’ mastery of care, depression and anxiety (Belle et al. 2006).

Services like “Telecare Scotland” enabled some carers to participate in paid employment, helping them to remain in a job that they might otherwise have had to give up (Beale et al. 2009). Approximately three-quarters of all the carers interviewed reported feeling ‘less stressed’. The benefit they most often cited was that this service offered them ‘peace of mind’ about the well-being and safety of the dependent person.

ICT-based services for informal carers also improved the quality of life of care recipients. ICTs for independent living (such as “Action”, “E-Care”, “Emergency Alarm”, “IPPI”, “Just Checking”, “Sophia”, and “Telecare Scotland”) and even those ICTs for the personal support and social integration of informal carers (“Action”, “Caring for Others”) improved care recipients’ health-related quality of life and their social lives. The most important improvements were: reduction of social isolation and increased sense of security, reduction of patient admissions to hospital or institutions, and improved subjective health status.

Specifically, “Action” mainly improved care recipients’ subjective quality of life, by reducing social isolation and giving them an increased sense of security (Bergström et al. 2010). “Caring for Others” reduced the time that patients spent in hospital or institutions. It also delayed admission into long-term care institutions (Marziali and Garcia 2011). In a survey of 400 users of “E-Care” made by independent researchers, a significant percentage (66.3 %) of older care recipients claimed to have achieved an improvement in their health status (Chiatti et al. 2011). In a 2006 representative survey of the “Emergency Alarm” service providers, there was a 40 % drop in the number of applicants for residential care and a 40 % fall in the number of days spent on rehabilitation in healthcare institutions in trials of this system (Chiatti et al. 2011). “Just Checking” reduced residential admission rates by 43 % (Herefordshire County Council and Primary Care Trust 2009). The main outcome of “Telecare Scotland” was the prevention of admissions to hospitals and rest homes (Newhaven Research 2011). According to service managers, “IPPI” enabled patients to maintain a social network (Sjölinder 2010; Östlund and Lindén 2011), and the personal assistance provided by voluntary godparents in “Sophia” contributed to a considerable improvement in the social lives of the care recipients.

The quality of care provided by informal carers and paid assistants also improved. In particular, ICTs for information and learning helped informal carers and paid assistants to improve their knowledge, skills and competences in caring. This result was the direct consequence of carers’ training programs and of access to informative materials. Evaluation data from “Action” revealed, for example, that family carers were better prepared to care for their spouses/partners at home (Hanson et al. 2011). “Campus”, according to its internal evaluation, appeared to increase job opportunities for migrant care workers, e.g. up to 70 % of the people trained and enroled in local professional registers obtained a job in one year (Chiatti et al. 2011).

In terms of the cost of care for end users (care efficiency and sustainability), we found that some initiatives were more affordable for individuals and households. ICT-based initiatives seem to be cost-effective if compared with similar ordinary care and support services. The “Caring for Others” service showed, in this respect, that personal support and social integration technology helped family carers to save money because they could access services remotely instead of travelling and participating in face-to-face support groups (Marziali and Garcia 2011).

ICT-based initiatives also helped carers to see their support services as more attractive (Acceptability). The main improvements were high levels of satisfaction, low drop-out rates, and high levels of willingness to use the ICT tool. These results were a direct consequence of solution providers taking into account user needs and perceptions in the design of the intervention, and the usability of interfaces and devices. Furthermore, ICT-based initiatives made carers more satisfied with the service, due to additional training, information and assistance given by service providers. “Action” undertook extensive usability and user acceptance work, based on a user-centred design model (Hanson et al. 2007). Users of “Campus” expressed high levels of satisfaction with the e-learning activities. The overall drop-out rate was extremely low (Chiatti et al. 2011). Carers of the “E-care” service indicated high levels of satisfaction with the service (Chiatti et al. 2011). In “Just Checking”, carers felt the system was very easy to instal, and it reassured them that their relatives were safe. It also helped them to respond quickly to a crisis situation (University of Nottingham and Nottinghamshire County Council 2010).

Regarding the impact on skills and infrastructures to access and use of ICT-based services (infrastructure and accessibility), major improvements reported were better access to and ease of use of technical devices to use different ICT-based materials (e.g. on-line and DVD) and education on digital skills and competences. These improvements were the direct consequence of specific interventions by solution providers to facilitate user access to support services. “Action” made efforts to make the service easily accessible, even for those with low digital skills (Magnusson et al. 2002). In “Cuidadoras en Red”, digital skills and competences were built up or improved through digital literacy sessions. The “Emergency alarm” service was previously assessed in order to adapt the service to different geographical and social contexts (Chiatti et al. 2011). “IPPI” was developed as a standardized tool that could be used with any kind of TV and phone (Östlund and Lindén 2011).

### Impact at meso-level (impact on families, companies and organizations, peer groups and care providers)

At the meso-level, some impacts on care recipients’ quality of life have notable benefits for efficiency in the use of social care and healthcare services (care efficiency and sustainability). The impact analysis identified savings to the care and health services as a result of improving the conditions of home care through ICT support to carers. Many initiatives (“Action”, “E-Care”, “Emergency Alarm”, “Telecare Scotland”, “Just Checking” and “Caring for Others”) attempted to estimate savings in various parts of the health and care system, by extrapolation from micro-data, or comparisons with alternative care scenarios. The results depended considerably on the local costs of conventional care, but the savings claimed were considerable.

For social care, reported impacts at the meso-level of the use of ICT-based services for informal care on care recipients’ quality of life included: delayed entry into institutional care for the older person, reduction in the number of formal home care visits and in overnight care stays, and improvements to the quality and effectiveness of formal care. For example, institutionalization was delayed in a large proportion of the families using “Action”, meaning a potential gross savings of over 200,000 Euros/year per family. In 2004, this service, which combined mainly information and learning and personal support and social integration technologies, made a savings of 10,000 Euros per year per family in the Borås municipality. This was the result of a reduction in the need for home help services and the delayed entry into a nursing home for many service users (Magnusson and Hanson 2005). In Hungary, in a 2006 representative survey, the use of the “Emergency Alarm” service reduced institutionalization both in residential and healthcare institutions by 40 % (Chiatti et al. 2011). “Just Checking” reduced the rates of admission to residential care by 43 % (Heredfordshire County Council and Primary Care Trust 2009).

In health care, the principal impacts were reduction in unplanned hospital admissions and in the length of hospital stays for care recipients (including reduced in-hospital rehabilitation). For instance, an evaluation made by independent researchers showed that “E-Care” reduced the number of admissions to hospitals and the number of users accessing hospital services by 50 % over a period of 2 years (Chiatti et al. 2011). “Telecare Scotland” increased early discharges from hospital among the 43,000 people who received the service over 5 years (Beale et al. 2009; Newhaven Research 2011).

Although the highest savings came from reduction in the use of institutional care, considerable savings also came from reducing home care services such as respite and care visits. There was evidence of cost savings from integrating independent living technologies in local care systems. The “Emergency Alarm” service and “Just Checking” made comparisons between ICT-based services and ordinary care services, confirming that besides delaying the need for admission to residential care, ICTs for independent living could help to reduce the number of home care visits (Heredfordshire County Council and Primary Care Trust 2009; Roworth-Gaun et al. 2009). In “Just Checking”, the costs of subscribing to the service (7–15 €/day) represented a considerable saving on the typical costs of a home-care assistant (44$/day) for someone with mild dementia (Department of Health 2008).

### Impact at macro-level (impact on the economic and social protection systems, and the health and social care systems)

Evidence of impact at the macro-level was much harder to find, as a few studies explored the savings that ICT-based services for informal carers and their paid assistants generated for the health and social care systems. These impacts were mainly due to the reduction of the need for help from formal care for both the carer and the older person. The ICT-based services reduced hospital admissions and the length of hospital stays. The service “Caring for Others” saved more than one million dollars (in one year) in the Province of Ontario, by improving care recipients’ quality of life and reducing the number of institutional admissions (Marziali and Garcia 2011). The “E-Care” initiative saved 600,000 Euros in the municipality of Bologna in 2 years, due to improved quality of life for care recipients and the reduced number of hospital admissions (Chiatti et al. 2011). From 2006 to 2011, “Telecare Scotland” estimated to have saved 78 million pounds (€93 millions) in Scotland, mainly by improving quality of life for care recipients and reducing both care home admissions and unplanned hospital admissions (Beale et al. 2009; Newhaven Research 2011).

Table 3 summarizes the main findings regarding the impacts at micro-, meso- and macro-level and highlights the different impacts by type of service.

**Table 3:** Impacts at micro-, meso- and macro-levels and per ICT-enabled services

Impact analysis (micro-level)			Impact analysis (meso-macro-level)	
Services	Impacts for informal carers	Impacts for older people	Social Services	Health Care
Independent living	(−) hours of care (−) eliminates the need for constant presence (+) peace of mind (−) anxiety (+) health-related quality of life (+) reconciliation of care and work and family	(+) independent living and delay dependency (+) health status (+) perception of safety (+) compliance in treatment (+) improved relation carer–older person	(−) number of care visits (−) overnight care stays Delays institutional care	(−) hospital admissions
Information & learning	(+) accessibility to training (+) finding and receiving appropriate information (+) caring skills and digital competence (+) employability (+) sense of security	(+) Quality of care	Delays institutional care (−) number of care visits (−) overnight care stays (+) quality and effectiveness of formal care	(−) hospital admissions (−) length of hospital stays
Personal support & social integration	(+) promotes development of informal social networks of carers (−) isolation (−) stress (+) Quality of life (+) reconciliation of care and work	(+) Quality of care (+) Quality of life (+) Improved relation carer–older person		
Care coordination	(−) stress (+) Quality of life (−) burden of care (+) reconciliation of care and work (+) builds trust with professionals	(+) Quality of care (+) Quality of life (+) health status	(−) number of care visits Delays institutional care	
			Savings of 79 m GBP (over 5 years), for 20 m GBP investment	

(−) means a decrease in the value of the variable, (+) means increase in the value of the variable. For example, (−) stress in the column of informal carers and paid assistants means that they informed of a decrease in stress due to the use of the ICT-based service. (+) quality in the column of older people means that they informed of a higher level of quality of life due to the use of the ICT-based service

## Discussion

The aim of this study was to explore, from a qualitative perspective, the benefits of ICT-based services for informal carers of older people and their paid assistants at micro-, meso- and macro levels, based on a cross-case analysis of documented impacts of implemented ICT-based services in the EU, the USA and Canada. The analysis demonstrates that these services can improve the quality of life of older people and carers alike, as well as enhancing their access to qualified long-term care. They also generate direct savings that contribute to the sustainability of care systems.

We found that ICT-enabled services for carers improve the quality of life of older adults and carers and the quality of care in different personal situations and welfare regimes. These services allow informal carers to maintain a better balance between care and work, improving their social lives and health, and helping them to remain active in the labour market. They also increase informal carers’ and paid assistants’ knowledge of caring, and its related skills and competences. Other studies have also pointed to the benefits of ICTs for informal carers’ health (Smith and Toseland 2006; Lam and Lam 2012) and care competences (Weiner et al. 2005; Buis 2008).

We saw that these benefits at the micro level also have an impact at meso- and macro-levels, specifically impacts on the efficiency of social care and health care services. The services analysed pointed to delayed entry into institutional care for older adults and a reduction in unplanned hospital admissions and the length of hospital stays. These reductions in the use of services could generate savings for health and social care systems. The meso- and macro-level benefits resulted partly from having more qualified and empowered carers who could accelerate the shift from institutional care to home care preference.

Nevertheless, this study also shows the lack of contrasted evidence on the impacts of ICT-based services for informal carers and paid assistants, mostly at meso- and macro-levels, as was also found by Morris et al. (2012) in a literature review of the 8,000 papers on smart technologies for older adults. The lack of reported knowledge about meso- and macro-benefits of ICT services may be slowing down their development and use, as it is needed in order to convince policy-makers to take appropriate decisions for investments. Future research should concentrate, therefore, on working to obtain more accurate data on the impacts of ICT-based services for carers at micro-, meso- *and* macro-levels in order to encourage public care services and systems to deploy these technologies as part of mainstream care delivery. Such encouragement is part of the long-term care strategy of European Policy (Social Protection Committee and European Commission, 2014). Studies on impacts among a greater number of ICT-based services as well as greater development and testing of the impact-assessment framework that was constructed for this study can aid practitioners and policy-makers in reaching this objective.

The findings highlighted in this paper need to be seen in light of some methodological limitations of this study. First, the methodological framework was not fully developed and tested during the project lifetime, being still the first attempt to build a comprehensive framework for assessing the impact of ICT-based initiatives in Europe. Such framework is clearly an added value of the project that provides a prototype of methodology to be further developed in the future and next directions of research in the field. Second, the 10 selected European initiatives are not a representative sample of all the operational ICT-based solutions carried out in Europe, but they bring an overview of relevant initiatives in the continent. The two interventions from North America are not representative of American and Canadian contexts. They were considered in this work because they can provide some insights on initiatives different from European ones and they present different approaches for impact assessment. Third, the cases were based on a limited number of interviews and existing documents, collected during a relatively short period of time in 2011, so they do not provide a fully comprehensive data base.

We can conclude that this first exploration at the impacts of the use of ICT-based services for informal carers and paid assistants showed that these services tend to be beneficial for their end-users, as well as for the governments as they can generate a more efficient use of services and more sustainable social protection systems. The findings can convince public authorities on the relevance to recognize the role of informal carers and their paid-assistants in their public long-term care service provision, as well as to deploy these services for their support. The research also provides to researchers insights on methodological issues for more accurate impact-assessment studies of these services for evidence-based policy.
